# Overexpression of the *Lolium perenne* L. delta1-pyrroline 5-carboxylate synthase (*LpP5CS*) gene results in morphological alterations and salinity tolerance in switchgrass (*Panicum virgatum* L.)

**DOI:** 10.1371/journal.pone.0219669

**Published:** 2019-07-16

**Authors:** Cong Guan, Yan-Hua Huang, Hui-Fang Cen, Xin Cui, Dan-Yang Tian, Yun-Wei Zhang

**Affiliations:** 1 College of Animal Science and Technology, China Agricultural University, Beijing, China; 2 Beijing Key Laboratory for Grassland Science, China Agricultural University, Beijing, China; 3 National Energy R&D Center for Biomass (NECB), Beijing, China; United States Department of Agriculture, UNITED STATES

## Abstract

In plants, Δ1-pyrroline- 5-carboxylate synthase (P5CS) is the rate-limiting enzyme in proline biosynthesis. In this study, we introduced the *LpP5C*S (*Lolium perenne* L.) gene into switchgrass by *Agrobacterium*-mediated transformation. The transgenic lines (TG) were classified into two groups based on their phenotypes and proline levels. The group I lines (TG4 and TG6) had relatively high proline levels and improved biomass yield. The group II lines (TG1 and TG2) showed low proline levels, severely delayed flowering, stunted growth and reduced biomass yield. Additionally, we used RNA-seq analysis to detect the most significant molecular changes, and we analyzed differentially expressed genes, such as flowering-related and CYP450 family genes. Moreover, the biomass yield, physiological parameters, and expression levels of reactive oxygen species scavenger-related genes under salt stress all indicated that the group I plants exhibited significantly increased salt tolerance compared with that of the control plants, in contrast to the group II plants. Thus, genetic improvement of switchgrass by overexpressing *LpP5CS* to increase proline levels is feasible for increasing plant stress tolerance.

## Introduction

Proline is synthesized predominantly from glutamate through two successive reductions catalyzed by Δ1-pyrroline-5-carboxylate synthase (P5CS) and Δ1-pyrroline-5-carboxylate reductase (P5CR) in the chloroplast and cytoplasm [1]. P5CS, as the rate-limiting enzyme, is controlled by feed-back inhibition and transcriptional regulation in proline synthesis [2]. Proline degradation occurs in mitochondria in which proline is converted back to glutamate by proline dehydrogenase (ProDH) and P5C dehydrogenase (P5CDH) [3]. A second pathway of proline synthesis is from ornithine, converting ornithine into proline [4, 5].

Proline has important roles under stress conditions, such as influencing cell proliferation, stabilizing membranes to prevent electrolyte leakage and maintaining normal concentrations of reactive oxygen species (ROS) [6]. In mitochondria, proline protects Complex II of the mitochondrial electron transport chain to stabilize mitochondrial respiration during salt stress [7]. Increased proline levels protect photosynthetic and antioxidant enzyme activities to alleviate salt stress in transgenic sorghum (*Sorghum bicolor* L.) [8]. Conversely, the *Atp5cs1* mutant shows reduced salt tolerance compared with that of the wild type (WT) under salt stress [9]. As a signaling molecules, proline and very-long-chain fatty acids (VLCFA) share dual roles in buffering the cellular redox status, and the products of their synthesis are useful for stress resistance [10]. Moreover, recent research shows that proline biosynthesis is the target of crosstalk between ABA signaling and phosphate homeostasis, and phosphate homeostasis is regulated via PHOSPHATE STARVATION RESPONSE 1 (PHR1) and PHR1- LIKE 1-mediated transcriptional activation of the *AtP5CS1* gene in *Arabidopsi*s [11]. Proline is also essential for protein synthesis and regulating plant development as a signaling molecule [12], with a crucial role demonstrated in the regulation of general protein synthesis and cell cycle transition in maize (*Zea mays* L.) [13]. Additionally, proline is involved in the flowering signal [14], and transgenic plants overexpressing *AtP5CS1* show early flowering and accumulate large amounts of proline under long- and short-day conditions [15]; *AtP5CS2*, a duplicated *AtP5CS* gene, is an early target of CONSTANS (CO), which is involved in flower transition [16].

Switchgrass is a perennial, warm season C4 model grass native to North America that is recognized as a dedicated bioenergy crop [17, 18]. China has a large area of unused marginal land, and consistent the principle of never competing with grain, such marginal land can be used to cultivate bioenergy crops [19]. Thus, salt resistance is a significant requirement for bioenergy crops. Unlike previous reports [20], our results show that transgenic switchgrass overexpressing *LpP5CS* had various phenotypes depending on their proline levels. The group I plants with higher proline content had high tolerance to salt stress and increased biomass yield, whereas the group II plants with lower proline content showed delayed flowering, salt sensitivity and reduced biomass yield. Additionally, our data of RNA-seq show that deficient expression of *P5CS* affected secondary metabolism and fatty acid metabolism pathways. Thus, further investigation of the mechanisms by which *LpP5CS* functions in multiple aspects of plant growth and in responses to abiotic stresses will help accelerate the breeding progress in plants.

## Materials and methods

### Plant materials and growth conditions

The lowland-type switchgrass cultivar Alamo was used for genetic transformation and salt tolerance improvement, which was maintained in a greenhouse under a 16 h light/8 h dark photoperiod at 25 ± 2 °C. The developmental stages of switchgrass were divided into five elongation stages (E1, E2, E3, E4 and E5) and three reproductive stages (R1, R2 and R3) [21]. When switchgrass grew to the R3 stage, the tillers, plant height, leaf width and length, stem diameter, internode number, internode length and panicle length of the transgenic and WT plants (T0) were measured. Each line had three biological replicates, and three tillers were measured in each biological replicate.

### Vector construction and plant transformation

*LpP5CS* (GenBank accession no. KC896627) was inserted into the pCAMBIA 1301 vector under the control of the *CaMV35S* promoter, and the other *CaMV35S* promoter was placed upstream of the selectable marker gene hygromycin phosphotransferase (*hph*) [22]. The pCAMBIA 1301-*LpP5CS* was introduced into the highly embryogenic callus of switchgrass (from a seed) using an *Agrobacterium*-mediated transformation method [23].

### PCR and Southern blot analysis of transgenic plants

Genomic DNA was isolated from putative transgenic plants following the modified 2×CTAB procedure [24]. The putative transgenic plants were identified by PCR with the specific *LpP5CS* primer (S1 Table). For Southern blot hybridization analysis, genomic DNA was digested overnight with the restriction enzyme *Sac*I and loaded in each lane. DNA blot hybridization using *hph* gene as probe was conducted according to standard procedure of the DIG Labeling and Detection starter kit II (Roche Applied Science, Mannheim, Germany), then the hybridized membranes were exposed to Kodak X-OMAT BT X-ray film for autoradiography [20].

### RNA isolation and quantitative real-time RT-PCR

Transcript levels of genes were measured using qRT-PCR. Total RNA from the leaves of transgenic and WT plants was isolated by the TRIzol reagent method (Invitrogen, Carlsbad, CA, USA). cDNA was synthesized from 1 μg of total RNA using a PrimeScript RT reagent Kit with gDNA Eraser (Takara, Shiga, Japan). qRT-PCR was conducted in an Eco48 Real-Time PCR System (Illumina, UK) according to the manufacturer’s instructions. The switchgrass *ubiquitin1 (Ubq1)* gene was used as the internal control [25], and the relative expression levels of genes were calculated using the 2^−ΔΔCT^ method [26]. Primers used for qRT-PCR are listed in S1 Table.

### RNA-seq analysis

Total RNA was extracted from transgenic and WT leaves at the E5 stage, and RNA samples included three groups: TG1 and TG2 lines (group II), TG4 and TG6 lines (group I), and WT1 and WT2 plants. The mature leaves of internode 3 (I3) from six independent plants were pooled for RNA extraction. The paired-end reads were aligned to the reference genome (http://phytozome.jgi.doe.gov/pz/portal.html#!info?alias=Org_Pvirgatum) using TopHat v2.0.12 [27]. HTSeq v0.6.1 [28] was used to count the reads numbers mapped to each gene. FPKM of each gene was calculated based on the length of the gene and reads count mapped to this gene. The DEGeq R package was used to determine the differential expression [29]. The RNA-seq data were uploaded in NCBI, with the NCBI Sequence Read Archive (SRA) number SRP130275.

### Preliminary evaluation of salt tolerance in transgenic and WT plants

Five transgenic (TG1, TG2, TG3, TG4 and TG6) and WT plants were subjected to a leaf senescence assay. Mature leaves from transgenic and WT plants (R1) were cut into 4 cm long pieces and soaked in 0 or 350 mM NaCl solution for 30 days [30]. Phenotypic changes were used to assess the effects of these treatments on the leaf pieces. Additionally, transgenic (TG1, TG2, TG3, TG4 and TG6) and WT plants were placed in 1/2× Hoagland nutrient solution supplemented with 350 mM NaCl for 7 days. Each line had three biological replicates, and three tillers were measured in each biological replicate.

### Salt-stress tolerance tests

Uniform tillers of both transgenic and control plants were chosen for the salinity stress test in sand culture. Transgenic and WT plants (R1) were treated with 1/2 × Hoagland nutrient solution supplemented with 0, 200, and 400 mM NaCl every 2 days. Plant height and leaf length of TG and WT were measured under salt stress on day 0 and day 30. Additionally, the fresh and dry weights of transgenic and WT plants under different NaCl concentrations (0, 200 and 400 mM) were measured on the 30^th^ day. Each line had three biological replicates, and three tillers were measured in each biological replicate.

### Physiological measurements

Relative water content (RWC) and electrolyte leakage (EL) were measured following previous protocol [31]. Malondialdehyde (MDA) was measured by the reaction of thiobarbituric acid (TBA) [32]. Proline was measured at 520 nm absorbance [33]. Chlorophyll, carotenoid, Na^+^ and K^+^ contents were measured following previous protocol [31]. Three plants from each independent line were used.

### Scanning electron microscopy

Leaf samples of 5-month-old transgenic and WT plants were used to measure the cell number. Additionally, leaf samples of transgenic and WT plants (R1) (0 and 400 mM NaCl salt stress) were used to measure the stomatal aperture. Tissue fixation was performed following previous protocol [34]. Microscopy analysis was conducted on an SEM (Hitachi S-3400N) using a 2 kV accelerating voltage, and the data were analyzed using Image-Pro Plus 6.0. Three plants from each independent line were used in the microscopy analysis.

### Histological analysis

Leaf fixation and embedding of the transgenic and WT plants (0 and 400 mM NaCl salt stress) were performed after salt treatment for 30 days [35]. Images were taken under an Olympus BX-51 compound microscope, and the data were analyzed using Image-Pro Plus 6.0. Three plants from each independent line were used in the histological analysis.

### Statistical analyses

Triplicate samples were collected for each transgenic and WT line. Data from each trait were subjected to analysis of variance (ANOVA). The significance of treatments was tested at the level of *P* < 0.05. Standard errors are provided in all tables and figures as appropriate. All statistical analyses were performed using the SPSS statistical software package (SPSS 20.0, IBM Company, USA).

## Results

### Molecular identification of transgenic plants

**Fig 1:**
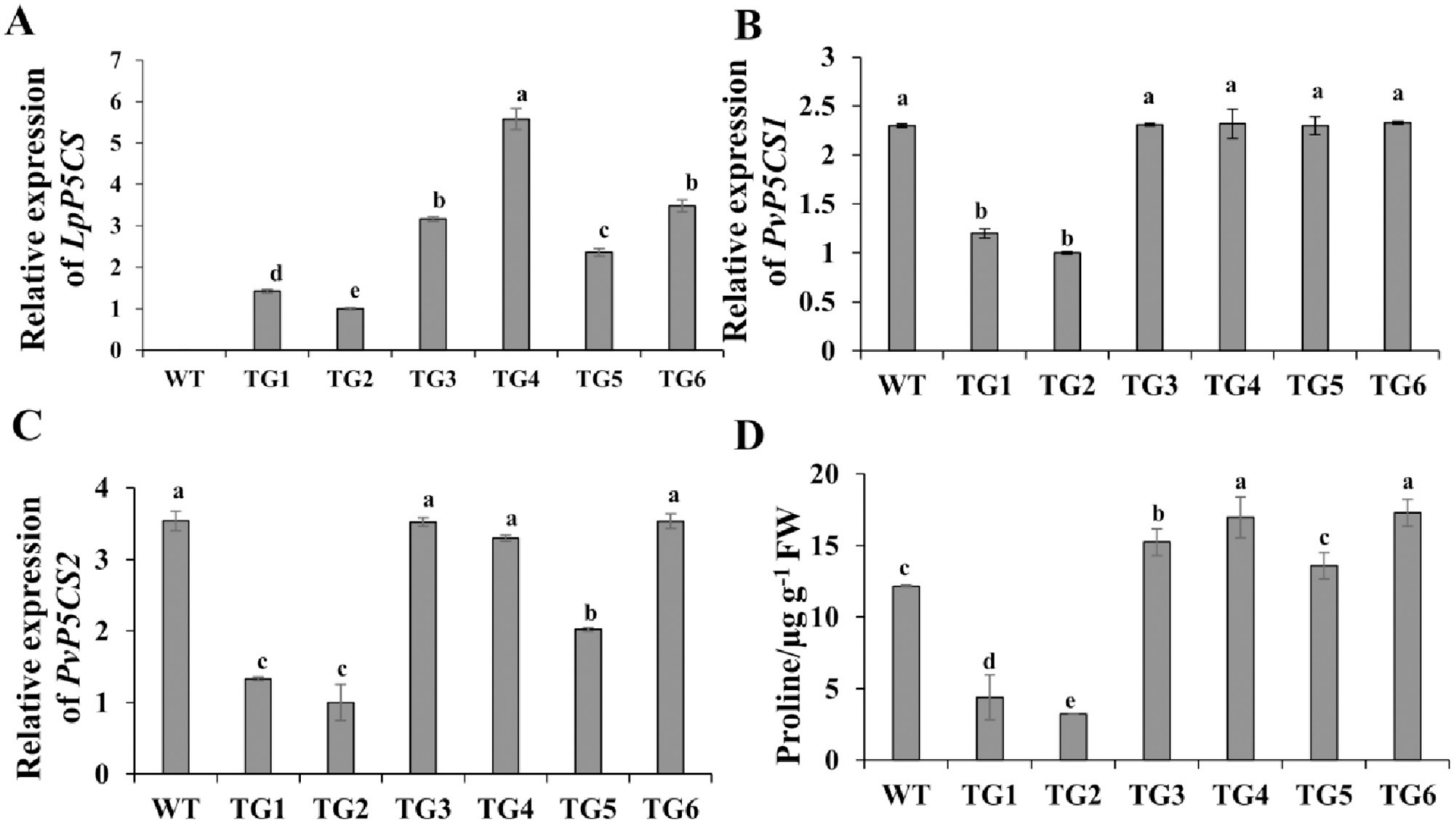
Molecular analysis of transgenic switchgrass lines overexpressing *LpP5CS*. (A) Relative expression levels of *LpP5CS* (TG2 was used as a control), (B) *PvP5CS1* and (C) *PvP5CS2* in transgenic and WT plants. (D) Proline content in transgenic plants. Switchgrass *Ubq1* was used as the reference for normalization, the relative expression levels of genes were calculated using 2^−ΔΔCT^ method, and the expression level of one plant (control) was defined “1”. Value are mean ± SE (*n* = 6). The significance of treatments was tested at the *P* < 0.05 level (one way ANOVA, Dunnett’s test).

In the DNAMAN software, homogeneous analysis indicated that the sequence similarity is 75.96% between *LpP5CS* and *PvP5CS1* gene and 83.97% between *LpP5CS* and *PvP5CS2* gene (S1 Fig). The hygromycin-resistant plants were screened by PCR using *LpP5CS* specific primers, and six transgenic lines were identified (S2 Fig). Additionally, the expression level of *LpP5CS* in TG1 and TG2 was lower than that in other transgenic plants (Fig 1A). The expression levels of the endogenous gene *PvP5CS* (*PvP5CS1* and *PvP5CS2*) in TG1 and TG2 were also lower than those in the WT, whereas those in TG4 and TG6 were unaffected (Fig 1B and 1C). Additionally, proline content was lower in TG1 and TG2 and higher in TG4 and TG6 than that in WT plants (Fig 1D).

### The effect of proline on plant growth and development

**Fig 2:**
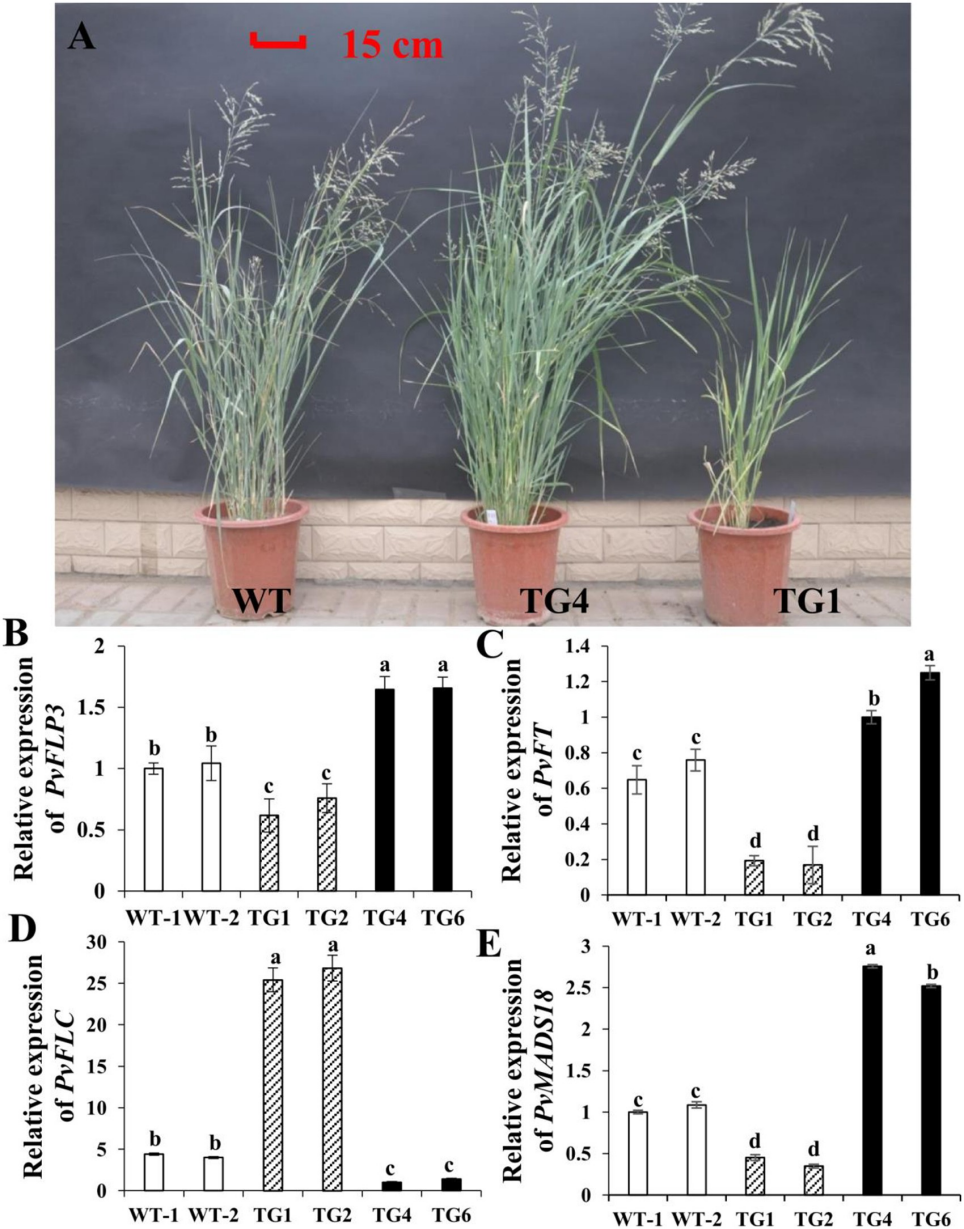
*LpP5CS* regulates growth and development in switchgrass. (A) Morphological characterization of transgenic switchgrass lines (5 months) overexpressing *LpP5CS*. (B-E) Relative expression levels of *PvFLP3*, *PvFT*, *PvFLC* and *PvMADS18* in transgenic plants and WT plants. Value are mean ± SE (*n* = 6). Switchgrass *Ubq1* was used as the reference for normalization. The significance of treatments was tested at the *P* < 0.05 level (one way ANOVA, Dunnett’s test). Leaf size is controlled by the complex coordination of cell division and expansion. To interpret the leaf size of group I, group II and WT plants, the leaf epidermal cells were viewed with scanning electron microscopy (SEM). The group II line (TG1) had a 36% reduction and the group I line (TG4) had a 43% increase in cell numbers compared with those of WT plants (Fig 3A–3C). We speculated that the increase in cell number was caused by increased expression of cell cycle-related genes. Thus, the expression level of cell cycle-related genes in TG1, TG4, and WT plants was analyzed. Cyclin-dependent kinases are the master regulators of the eukaryotic cell cycle to stimulate cell division and tissue growth. Therefore, we measured the expression level of the D-type cyclin gene (*PvCYCD*) (Pavir.J14518.1) and B-type cyclin gene (*PvCYCB*) (Pavir.J38704.1). The results showed that the expression levels of *PvCYCD* and *PvCYCB* were significantly up-regulated in the TG4 plants and significantly down-regulated in the TG1 plants compared with those in WT plants (Fig 3D). Collectively, these findings indicate that proline is crucial to plant growth and development.

Based on the proline content and morphological characterization, TG4 and TG6 lines were assigned to group I, and TG1 and TG2 lines were assigned to group II. To evaluate the effect of proline on plant growth and development, we measured the internode number, internode length, plant height, leaf blade length and width, internode diameter, and tiller number. The group I lines (TG4 and TG6) showed no significant difference in internode number, internode diameter, or leaf length compared with those in WT plants but did show significant differences in plant height, internode length, tiller number, spike length and leaf blade width (Table 1). By contrast, the group II lines (TG1 and TG2) had low levels of proline and showed stunted growth. Compared with those of WT plants, the group II lines showed an average of 55.5, 35 and 54% reduction in plant height, internode diameter and tiller number, respectively. Therefore, the reduced plant height of group II lines was caused by shortened internodes, with the uppermost internode of group II lines significantly shorter than that of the WT. Additionally, the group II lines exhibited late flowering (Fig 2A), which led us to speculate that the expression of related floral induction genes was inhibited. Thus, we measured the expression levels of flowering-related genes, such as flowering-promoting factor 1-like protein 3 (*PvFLP3*), FLOWERING LOCUS C (*PvFLC*), FLOWERING LOCUS T (*PvFT1*) and *PvMADS18*. The expression levels of *PvFLP3*, *PvFT* and *PvMADS18* were higher in the group I lines and lower in the group II lines than those in WT plants, and the expression levels of *PvFLC* in group II lines were higher than those in group I and WT plants (Fig 2B–2E), suggesting that proline increased floral induction by improving the expression of *PvFLP3*, *PvFT* and *PvMADS18* and inhibiting the expression of *PvFLC* in switchgrass.

**Table 1:** The characterization of growth and development in transgenic and WT plants.

	Internode number	Internode length(cm)	Plant height(cm)	Leaf blade length(cm)	Leaf blade width(mm)	Spike length(cm)	Internode diameter(mm)	tiller number
**WT**	5±0a	18.1±0.3b	104.5±0.9b	50.7±1.0c	12.3±0.5c	38.4±2.1b	3.24±0.11c	23±1.3b
**TG1**	4±0a	12.7±0.5c	64.6±0.7c	40.2±0.7d	9.9±0.3d	0.0±0.0c	2.40±0.05d	12±0.6c
**TG2**	3±0a	7.8±0.3d	50.4±0.7d	34.3±0.5e	9.1±0.2e	0.0±0.0c	2.26±0.07e	11±0.4c
**TG4**	5±0a	20.5±0.4a	125.4±0.6a	60.7±0.5b	13.1±0.2b	43.2±0.7a	3.80±0.05a	35±1.8a
**TG6**	5±0a	21.3±0.5a	128.2±0.7a	67.0±0.4a	13.9±0.2a	44.7±0.5a	3.55±0.04b	33±2.1a

Internode number, internode length and internode diameter (internode 3), plant height, leaf blade length and width, spike length and tillers were measured in transgenic and WT switchgrass after 5-month growth in the greenhouse. Each line had three biological replicates, and three tillers were measured in each biological replicate. Value is mean ± SE (*n* = 3) in tiller number, and the other value are mean ± SE (*n* = 9). The significance of treatments was tested at the *P* < 0.05 level (one way ANOVA, Dunnett’s test).

**Fig 3:**
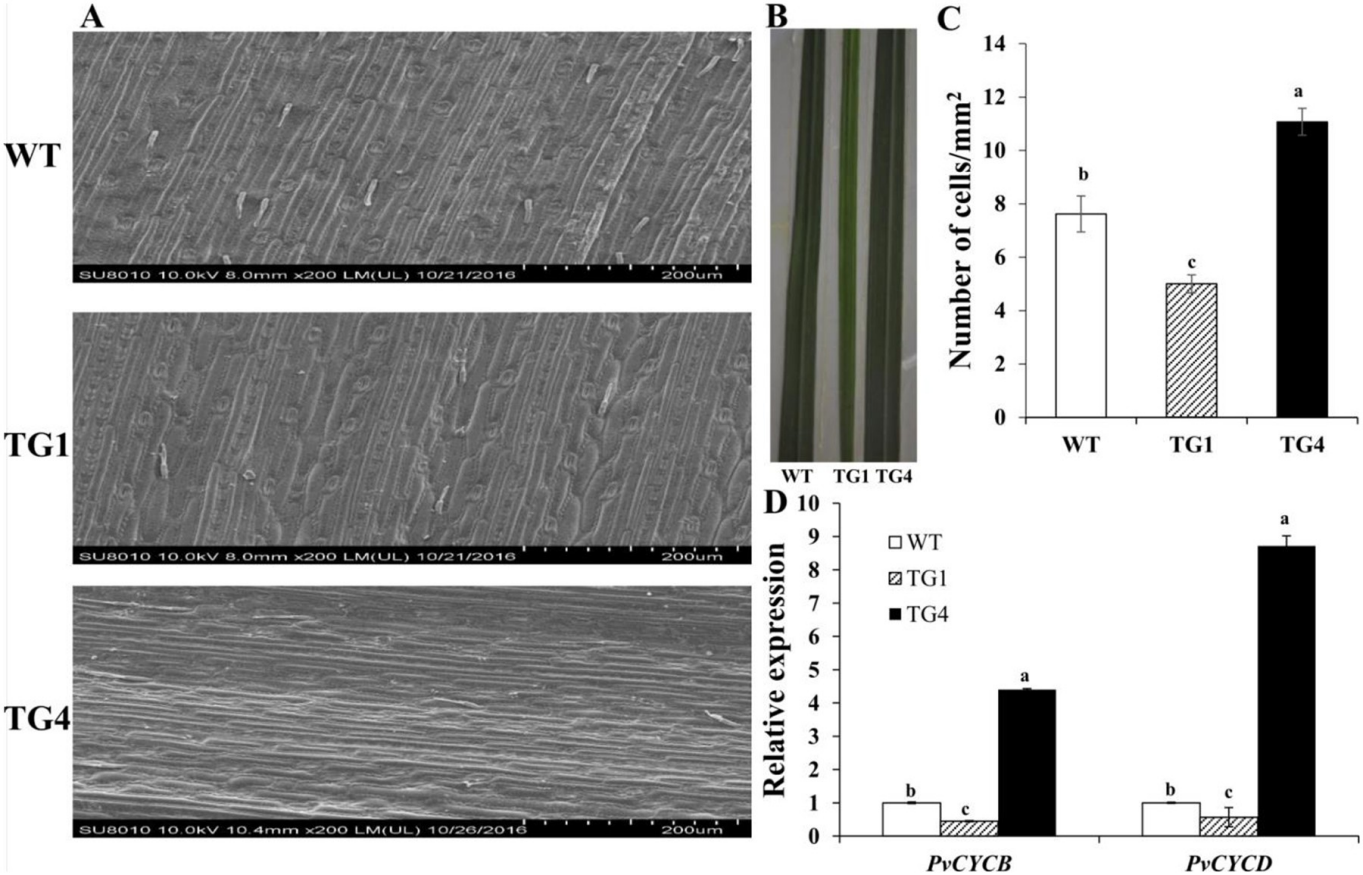
*LpP5CS* regulates cell size. (A) Scanning electron micrograph (SEM) imaging cells on the adaxial surface in the leaf of TG1, TG4 and WT. Bar = 200 um. (B) Leaf width and (C) cell number in TG1, TG4 and WT. Value are mean ± SE (*n* = 3). (D) Relative expression levels of *PvCYCD* and *PvCYCB* in transgenic plants and WT plants. Value are mean ± SE (*n* = 6). Switchgrass *Ubq1* was used as the reference for normalization. The significance of treatments was tested at the *P* < 0.05 level (one way ANOVA, Dunnett’s test).

### RNA-seq analysis

**Fig 4:**
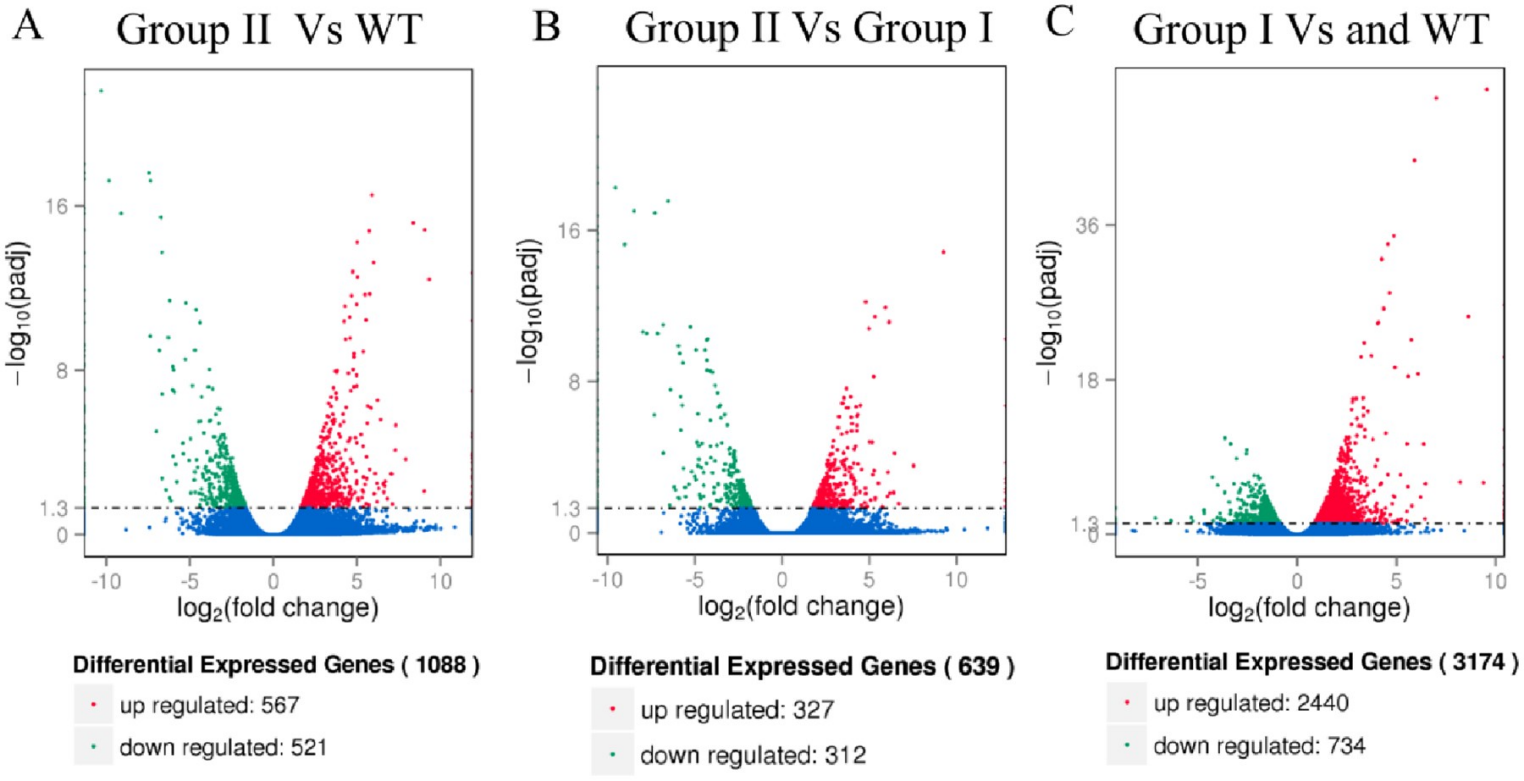
RNA sequencing of the group I, group II and WT plants. The volcano plot: (A) Group II Vs WT lines, (B) Group I Vs Group II lines, (C) Group I Vs WT lines.

In summary, 71.03, 71.35 and 70.36 million raw reads were generated from control samples (WT1 and WT2 lines), group II transgenic samples (TG1 and TG2 lines) and group I transgenic samples (TG4 and TG6 lines), respectively. Additionally, the average Q20 and Q30 levels and GC-rich contents of the six samples were 98.07, 95.29, and 54.14, respectively (S2 Table). In the group II lines, 327 genes were up-regulated and 312 genes were down-regulated compared with the group I lines, and 567 genes were up-regulated and 521 genes were down-regulated compared with WT plants. Compared with WT plants, 2440 genes were up-regulated and 734 genes were down-regulated in the group I lines (Fig 4). Among differentially expressed genes, the most significantly up- and down-regulated genes (log_2_Fold Change > 2) are listed in S3 and S4 Tables, respectively. To confirm the accuracy and reproducibility of the Illumina RNA-seq, 12 DEGs were selected to perform a correlation analysis. The results showed that RNA-seq and qRT-PCR were significantly correlated (R^2^ = 0.9838), indicating the reproducibility and reliability of the RNA-seq data (S3 Fig).

GO and KEGG enrichment information is included in S5 and S6 Tables, which showed that secondary metabolism and fatty acid metabolism pathways had significant differences among the group I, group II and WT plants. Thus, we measured the expression levels of related differentially expressed genes by qRT-PCR. CYP450 family genes are involved in many metabolic pathways. In our RNA-seq results, the differentially expressed genes included *PvCYP88A*, *PvCYP714B3*, *PvCYP72A*, *PvCYP94A1* and *PvCYP86A2*, which are involved in GA biosynthesis, secondary metabolism and fatty acid metabolism pathways, and the expression levels of those genes were up-regulated in the group I lines and down-regulated in the group II lines compared with those of WT plants (S4 Fig). These findings suggest that proline affects a series of metabolic pathways through cytochrome P450 genes in switchgrass. The differentially expressed transcription factors (TFs) (Corrected *P*-value < 0.05) are listed in S7 Table, and the identified 224 transcription factors belong to 43 TF families, primarily containing abiotic stress, floral induction, and growth and development related genes. Notably, MYB (20, 8.93%) was the most abundant, followed by WRKY (17, 7.59%), SNF2 (16, 7.14%), bHLH (15, 7.70%), and Orphans and NAC (14, 6.25%) (S5 Fig).

### Preliminary evaluation of salt tolerance in transgenic and WT plants

To test whether overexpression of the *LpP5CS* gene improved the salt tolerance of transgenic plants, the extent of damage (bleaching) under salt stress in transgenic leaves was visualized in a preliminary *in vitro* investigation of salt stress tolerance. The leaves of transgenic (TG1, TG2, TG3, TG4 and TG6) and WT plants all remained green under 0 mM NaCl. However, the leaves of TG1, TG2 and the WT exhibited excessive bleaching on the 20^th^ day under high salt stress (350 mM NaCl), and the extent of damage (bleaching) in TG1 and TG2 was more serious than that in the WT; by contrast, the leaves of other transgenic lines remained green (S6A Fig). Additionally, transgenic lines were treated with a 350 mM NaCl solution for a week to test their responses to salt shock. As shown in S6B Fig, TG3, TG4 and TG6 were most resistant to salt shock, whereas the leaves of TG1, TG2 and the WT showed obvious wilting. We also measured the relative water content (RWC) and electrolyte leakage (EL) under salt stress. Similar to the results of salt injury symptoms, TG3, TG4 and TG6 had higher water content and lower electrolyte leakage than those of WT plants; by contrast, TG1 and TG2 lost water more quickly and had higher electrolyte leakage than that of WT plants (S6C Fig and Fig 6D). Thus, TG1 and TG4 were chosen for detailed salt-tolerance tests.

### Salt stress test of transgenic plants

#### Growth performance under salt stress

**Fig 5:**
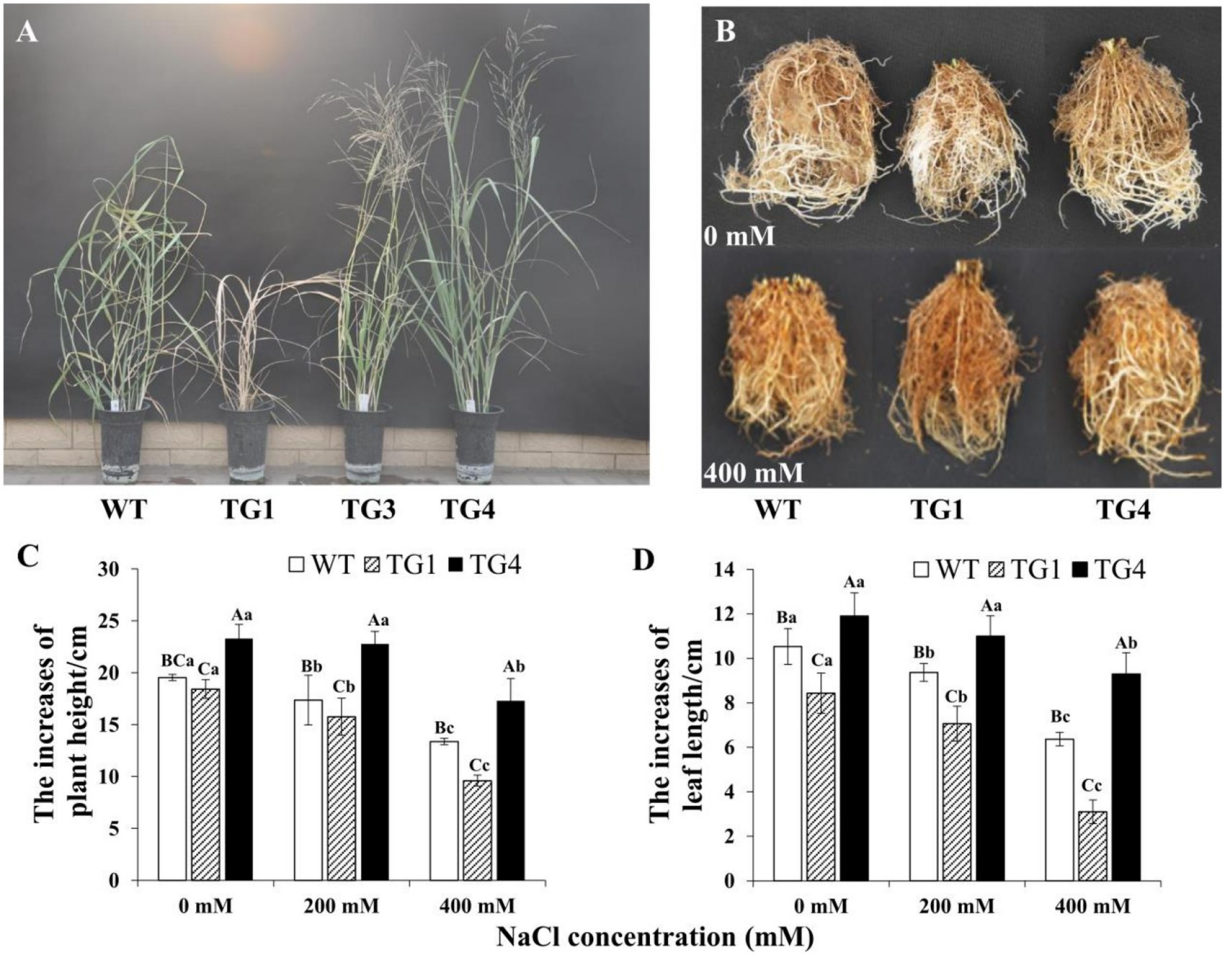
The salt tolerant evaluation of TG1, TG4 and WT plants. (A) Phenotypic characterization of TG1, TG4 and WT plants under 400 mM NaCl treatment on the 30^th^ day. (B) Roots of TG1, TG4 and WT under 0 and 400 mM NaCl on the 30^th^ day. (C) The increases of plant height, (D) the increases of leaf length in TG1, TG4 and WT under 0, 200 and 400 mM NaCl treatment for 30 days. Value are mean ± SE (*n* = 9). The significance of treatment (0, 200 and 400 mM NaCl concentration) an sample type (WT and transgenic plants) was tested at the *P* < 0.05 level (two way ANOVA), capital letter represents the difference between WT and transgenic plants under the same salt concentration, lowercase represents the difference of WT or transgenic plants under 0, 200 and 400 mM NaCl concentration.

TG1 displayed stunted growth and fast wilting, whereas TG4 remained upright and showed obviously early flowering under salt stress (Fig 5A). Additionally, TG1, TG4 and the WT all had new roots when grown under the normal condition; however, under 400 mM NaCl, the roots of TG1 were the darkest, and the TG1 plants almost died, whereas TG4 plants remained with new roots, and the color of roots was the lightest compared with that of WT roots (Fig 5B). Thus, the damage to roots ultimately led to shoot death in the TG1 plants under 400 mM NaCl. To better understand the growth of TG1, TG4 and the WT, we measured the increases in plant height and leaf length under salt stress. The increases in plant height and leaf length were the lowest in TG1, followed by those in the WT, and the increases were the highest in TG4 under salt stress (Fig 5C and 5D). Additionally, we also measured the fresh and dry weights of TG1, TG4 and the WT under salt stress, and the fresh and dry weights were both the lowest in TG1, followed by those in the WT, and the highest increases were in TG4 under salt stress (S7 Fig), indicating that *LpP5CS* positively regulates salt resistance in switchgrass.

#### The physiological response of the transgenic switchgrass under salt stress

**Fig 6:**
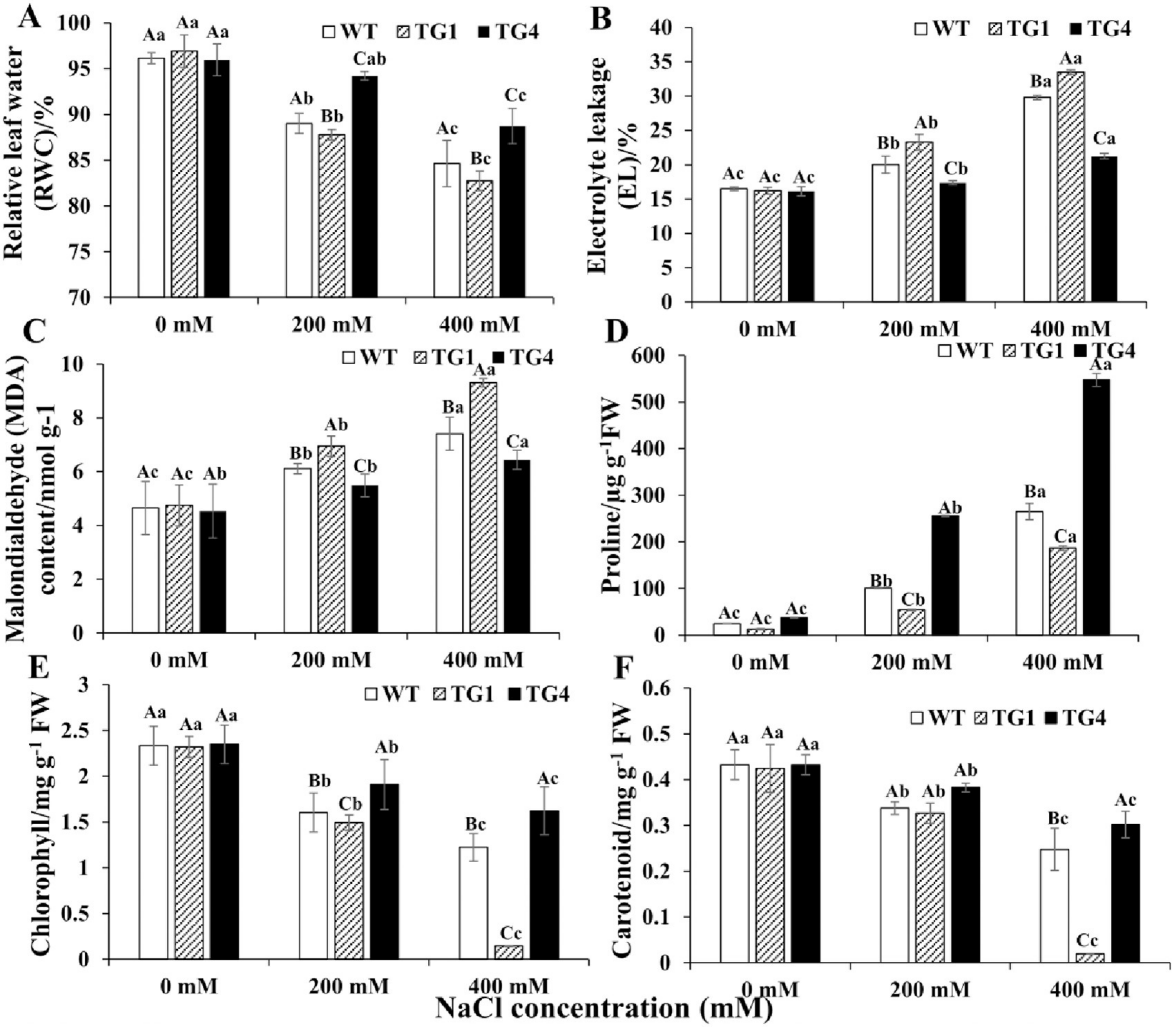
Physiological analysis of TG1, TG4 and WT plants under salt stress (0, 200 and 400 mM NaCl). (A) RWC, (B) EL, (C) MDA, (D) proline, (E) total chlorophyll (chlorophyll A + B), (F) carotenoid in TG1, TG4 and WT under salt stress. Value are mean ± SE (*n* = 3). The significance of treatment (0, 200 and 400 mM NaCl concentration) an sample type (WT and transgenic plants) was tested at the *P* < 0.05 level (two way ANOVA), capital letter represents the difference between WT and transgenic plants under the same salt concentration, lowercase represents the difference of WT or transgenic plants under 0, 200 and 400 mM NaCl concentration.

**Fig 7:**
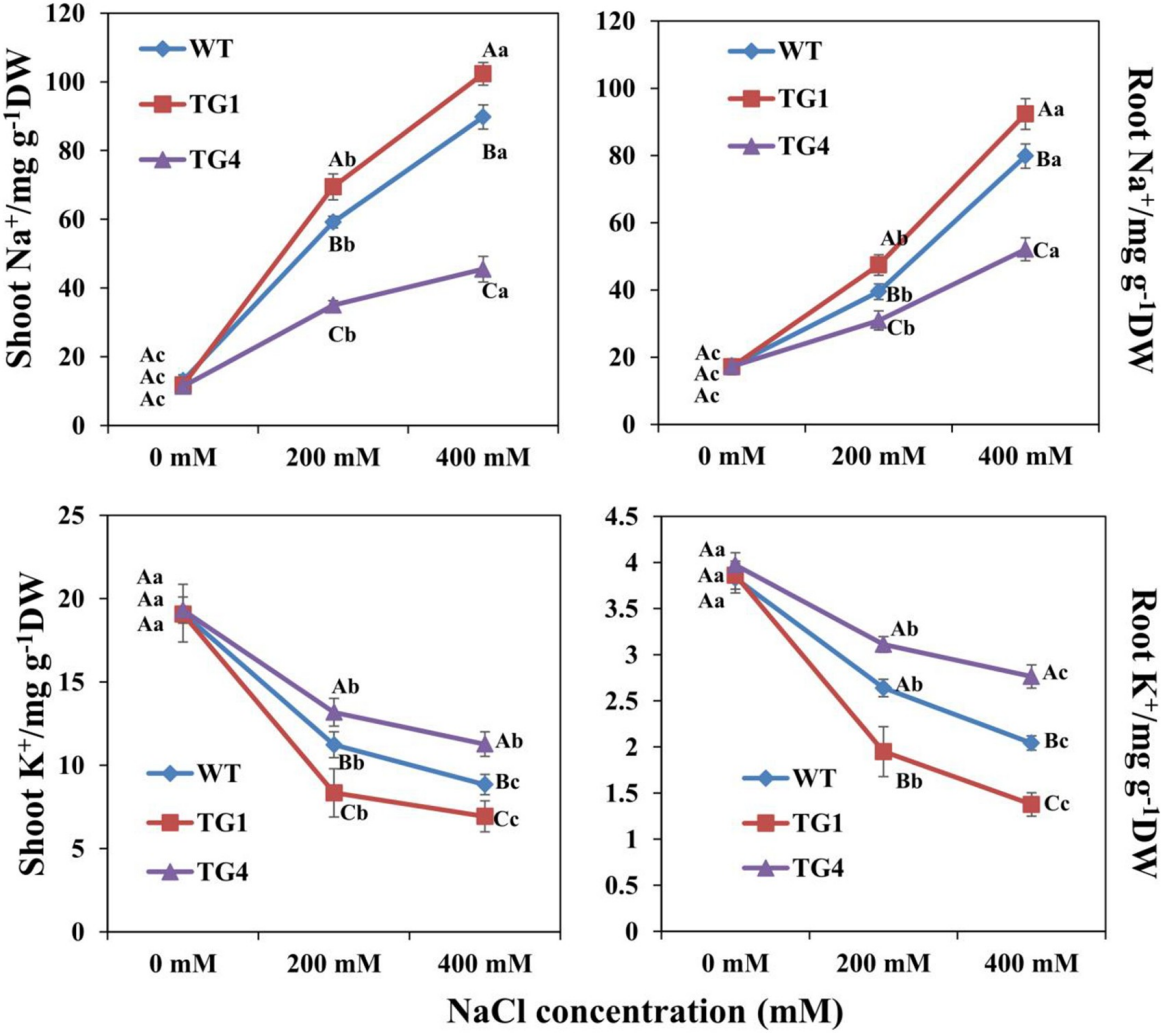
Na^+^, K^+^ contents of TG1, TG4 and WT plants under salt stress (0, 200, and 400 mM NaCl). Value are mean ± SE (*n* = 3). The significance of treatment (0, 200 and 400 mM NaCl concentration) an sample type (WT and transgenic plants) was tested at the *P* < 0.05 level (two way ANOVA), capital letter represents the difference between WT and transgenic plants under the same salt concentration, lowercase represents the difference of WT or transgenic plants under 0, 200 and 400 mM NaCl concentration.

To determine the water deficit response, relative water content (RWC) was determined for detached leaves under salt stress (0, 200, 400 mM NaCl). All plants had similar leaf water content under the normal condition; however, the TG1 line lost water faster than the WT, whereas the TG4 line lost water more slowly under 200 and 400 mM NaCl. Compared with the normal condition, the relative water content of TG1 and the WT decreased by 15 and 12%, respectively, whereas that of TG4 only decreased by 7% under 400 mM NaCl (Fig 6A). Salt stress can cause damage to plant cells, which leads to leakage of cellular electrolytes, and electrolyte leakage (EL) is an indicator for membrane damage. In our results, EL and malondialdehyde (MDA) content were similar in TG1 (group II), TG4 (group I) and the WT under 0 mM NaCl, but under 400 mM NaCl, both were the highest in TG1, followed by those in the WT, with EL and MDA content the lowest in TG4 (Fig 6B and 6C). Chlorophyll and carotenoid concentrations were similar in TG1 (group II), TG4 (group I) and the WT under the normal condition. However, under 400 mM NaCl, in TG1, chlorophyll and carotenoid concentrations were 0.14 and 0.02 mg/g, respectively, whereas in TG4, concentrations were the highest and 10- and 16-fold greater than those in TG1, respectively (Fig 6E and 6F), suggesting that TG4 plants were less prone to chlorophyll degradation and maintained a higher photosynthesis capacity than that of WT plants under salt stress. Additionally, no significant differences were observed in Na^+^ and K^+^ contents in both shoots and roots under the normal condition (Fig 7). However, TG1 accumulated more Na^+^ and less K^+^ and TG4 accumulated less Na^+^ and more K^+^ in both shoots and roots compared with concentrations in the shoots and roots of WT plants at 400 mM NaCl (Fig 7). Not surprisingly, the proline content of TG4 was the highest under various salt stress conditions and was 3.0- and 2.1-fold greater than that in TG1 and the WT under 400 mM NaCl, respectively (Fig 6D). These findings all indicate that *LpP5CS* confers salt tolerance in transgenic switchgrass.

#### Histological analysis and scanning electron micrograph (SEM)

**Fig 8:**
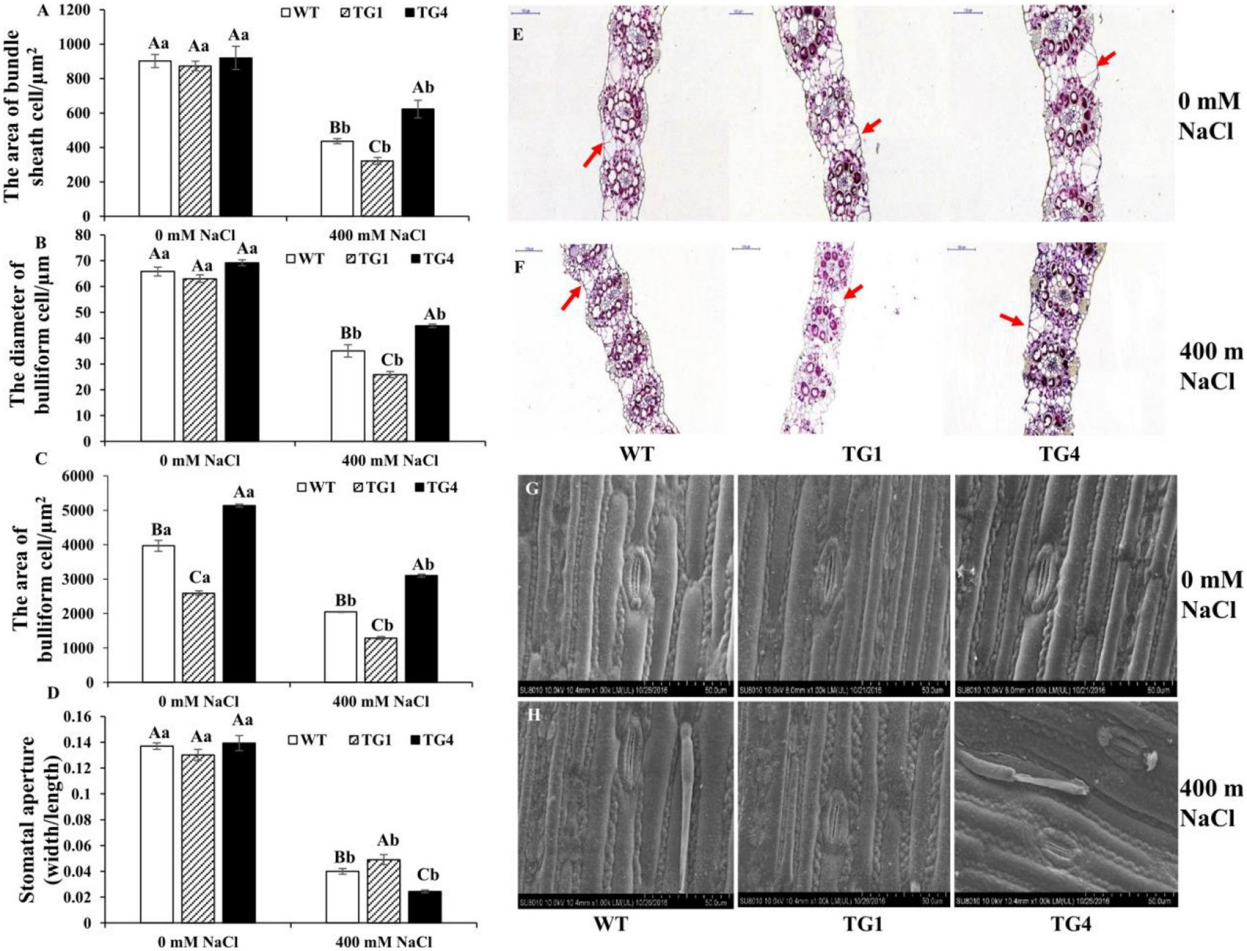
Histological analysis of the leaf in TG1, TG4 and WT plants under 0 and 400 mM NaCl. (A) The areas of bundle sheath cells, (B) the diameter of bulliform cells, (C) the areas of bulliform cells, (D) the stomatal aperture of the transgenic and and WT under 0 and 400 mM NaCl. Histological analysis in the leaf of internode 3 of TG1, TG4 and and WT under 0 (E) and 400 mM NaCl (F), Bar, 100 um. Scanning electron micrograph (SEM) imaging cells on the adaxial surface in the leaf of internode 3 of TG1, TG4 and WT under 0 (G) and 400 mM NaCl (H), Bar, 50.0 um. Value are mean ± SE (*n* = 3). The significance of treatment (0, 200 and 400 mM NaCl concentration) an sample type (WT and transgenic plants) was tested at the *P* < 0.05 level (two way ANOVA), capital letter represents the difference between WT and transgenic plants under the same salt concentration, lowercase represents the difference of WT or transgenic plants under 0 and 400 mM NaCl concentration.

An increase in the bulliform cell volume can store more water to facilitate leaf unfolding and improve salt tolerance in plants [36]. Thus, we measured the areas of bundle sheath cells and the diameters and areas of bulliform cells. The areas of bundle sheath cells were similar in TG1, TG4 and the WT under the normal condition (Fig 8A); whereas the areas of bulliform cells were significantly different among the TG1, TG4 and WT plants under the normal condition, with the largest in TG4 (Fig 8C). Under 400 mM NaCl, the areas of bundle sheath cells and the diameters and areas of the bulliform cells all showed huge decreases in TG1, TG4 and WT plants, but the smallest decrease was in TG4 and the largest in TG1 plants (Fig 8A–8C and 8F). Additionally, we assumed that TG4 could limit water loss to improve its salt tolerance through stomatal closure. To confirm this assumption, we measured the stomatal apertures in the leaves of TG1, TG4 and the WT under 0 and 400 mM NaCl. Under the normal condition, the stomatal apertures were similar in TG1, TG4 and WT plants, whereas the stomatal aperture of TG4 was the smallest, followed by that of the WT and that of TG1 was largest under 400 mM NaCl (Fig 8D, 8G and 8H). Thus, these results confirmed our assumption that TG4 plants could hold more water in its bulliform cells and limit water loss through stomatal closure to improve salt tolerance.

## Discussion

Notably, the expression levels of *P5CS* and proline content were significantly different among the transgenic plants (Fig 1). The *CaMV 35S* promoter can cause transgene silencing in some cases [37]. The growth of TG1 and TG2 was abnormal, and they exhibited hypersensitive response to salt stress; whereas the cassette of 35S::*LpP5CS* was functional in TG4 and TG6, and the two transgenic plants had increased proline content and salt tolerance.

### The effect of proline on phenotypic characteristics of the transgenic plants

Group II plants grew the slowest and had the latest flowering time (Fig 2A). Switchgrass plants are estimated to require a high proline level to accelerate the growth and change the flowering time. The result from *Atp5cs2* T-DNA insertion mutants also indirectly support this hypothesis, because mutants showed reduced proline content, stunted growth, and a delayed floral transition [38]. Thus, proline has an important role in plant growth and floral transition. On one hand, as outcrossing species, pollen-mediated transgene flow is a major concern for field release of transgenic bioenergy crops [39]. Thus, the early flowering of group I lines could reduce the pollen-mediated flow with WT plants, which would minimize the risk of cross pollination between transgenic and WT plants. On the other hand, early flowering could also shorten growth cycle and increase mowing times.

The group I lines with increased expression levels of *P5CS* and proline content showed increased biomass. The number of cells was highest in TG4 (Fig 3C), and the expression levels of cell cycle-related genes (*PvCYCD* and *PvCYCB*) were significantly up-regulated in TG4 (Fig 3D). Thus, an increase in the level of proline may improve the expression of cell cycle-related genes and affect the coordination of cell division and cell expansion, which lead to a change in biomass. Many similar studies support this hypothesis. The antisense *AtP5CS* transgenic Arabidopsis show morphological alterations in leaves and a defect in elongation of inflorescences, and these phenotypes are suppressed by proline, because proline specifically affects structural proteins of cell walls [40]. *NtProDH* antisense transgenic tobacco cells show more active cell division than that of WT cells due to an increase in proline content [41]. The 35Sp-*HvProT* transgenic Arabidopsis plants have less proline accumulation, and the addition of exogenous L-proline restores the impaired growth of transgenic plants [42]. Additionally, proline deficiency suppresses the transcriptional levels of major cyclin genes, causing cell cycle arrest and a reduction in cell proliferation in maize mutants, demonstrating that proline plays a crucial role in the regulation of the cell cycle transition [13].

### RNA-seq analysis in transgenic switchgrass

The *P5CS1* promoter contains sequence motifs that are conserved in related Brassicaceae species and can be binding sites for bZIP, MYB, MYC, AP2/ERBP, and C2H2_Zn type transcription factors [1]. In our results, in addition to bZIP, MYB, AP2/ERBP, and C2H2_Zn type transcription factors, we found transcription factors of differentially expressed transcripts in the two transgenic groups and WT switchgrass also included AUX/IAA and ARF (S5 Fig). In our results, the KEGG enrichment showed that secondary metabolism and fatty acid metabolism pathways had significant differences among the group I, group II and WT plants, with some CYP450 family genes involved in those metabolic pathways [43–51]. Notably, functional analysis of conserved CYP domains shows that they have a proline-rich hinge region [49], suggesting that CYP450 family genes have connection with proline. Thus, we speculated that proline affected a series of metabolic pathways through CYP450 genes in switchgrass, but the specific regulatory mechanisms require further study. Additionally, *FLP3*, *MADS18*, *FLC* and *FT* are all involved in the regulation of floral induction [52, 53], and the expression levels of these genes showed significant differences among the group I, group II and WT plants. *CO* is involved in proline biosynthesis to regulate the flowering phenomenon, and *CO* and floral repressor *FLC* both encode MADS-box transcription factors and regulate *FT* and *SOC1* to influence flowering [16, 54]. Thus, we speculated that those genes were involved in proline biosynthesis to regulate floral induction.

### The effect of proline on salt tolerance in transgenic switchgrass

Salt tolerance can be estimated from growth performance. Transgenic switchgrass overexpressing vacuolar Na^+^ (K^+^)/H^+^ antiporter gene (*PvNHX1*) had higher proline content and dry weight than those of control plants under salt stress [55]. Transgenic chickpea overexpressing *P5CSF129A* had increased proline accumulation, and root biomass of transgenic plants increased under both salt and drought stress conditions. The authors speculated that proline may play an indirect role in increasing root biomass in the transgenic plants under stress conditions [56]. Additionally, a study shows that increases in chlorophyll and carotenoid levels increase salt tolerance and biomass in transgenic sweetpotato [57]. In our results, the fresh and dry weights of TG4 plants were the highest (S7 Fig), and the total chlorophyll and carotenoid contents of TG4 were also the highest under 400 mM NaCl (Fig 6E and 6F). Thus, proline may reduce chlorophyll degradation to maintain a high capacity for photosynthesis, which might be one of the reasons why TG4 plants had the highest biomass under salt stress. Plants accumulate more Na^+^ and absorb less K^+^ under salt stress, causing an imbalance in cellular homeostasis, and the accumulation of Na^+^ in the cells and tissues of plants adversely affects growth and development [58], such as lateral root development and root hair formation [55, 59]. Therefore, maintaining K^+^ levels and minimizing Na^+^ uptake are important to the survival of plants under salt stress. Additionally, an obvious correlation is observed between increases in proline contents and the tight control of Na^+^ uptake in *Thellungiella halophila* under salt stress [60]. In our results, the roots and shoots of TG1 had higher Na^+^ contents and lower K^+^ contents than those of the WT (Fig 7), with almost no new roots and shoots that were withered and yellow under salt stress (Fig 5A and 5B), in contrast to TG4 plants. Thus, proline plays an important role in maintaining cellular homeostasis to reduce the damage to plant growth and development under salt stress.

Under salt stress, ROS cause oxidative damage to macromolecules and cell structures. Proline can affect ROS scavengers to minimize ROS damage under salt stress. Transgenic sugarcane (*Saccharum officinarum*) overexpressing the *VaP5CS* gene has increased levels of proline and reduced levels of MDA under salt stress. In our study, TG4 also had higher proline and lower MDA levels compared with WT plants when subjected to salt stress, whereas TG1 plants did not show a similar response (Fig 6C and 6D) [61]. Generally, high proline levels change the ROS homeostasis in living cells by affecting ROS-mediated signal pathways, and ultimately improve salt tolerance [62]. *AtP5CS1* gene improves salt tolerance by activating the ABA signal pathway, and promotes the accumulation of endogenous ABA. ABA is an important regulator of the stomatal closure to reduce the transportation of Na^+^ from roots to shoots, additionally, to minimize water loss via transpiration in plants [63, 64]. In our results, the relative water content was the highest in TG4, followed by the WT, and was the lowest in TG1 (Fig 6A). Recent research shows that transgenic plants overexpressing *BdPP2CA6* had increased stomatal closure to conserve water, which contributed to improved salt tolerance [65]. Similarly, in our results, the stomatal aperture of TG4 was the lowest, whereas that of TG1 was the highest (Fig 8D); thus, TG4 had increased stomatal closure to conserve water and maintain physiological functions under salt stress. Additionally, increasing the bulliform cell volume can store more water to facilitate leaf unfolding and improve salt tolerance in plants [36]. In our results, the areas of bundle sheath and bulliform cells in TG4 were greater than those in TG1 and the WT under salt stress. These reasons might explain why the TG4 plants had higher relative water content than that in TG1 and the WT under salt stress, suggesting that *LpP5CS* contributes to improve salt tolerance by increased retention of water in switchgrass.

## Conclusions

**Fig 9:**
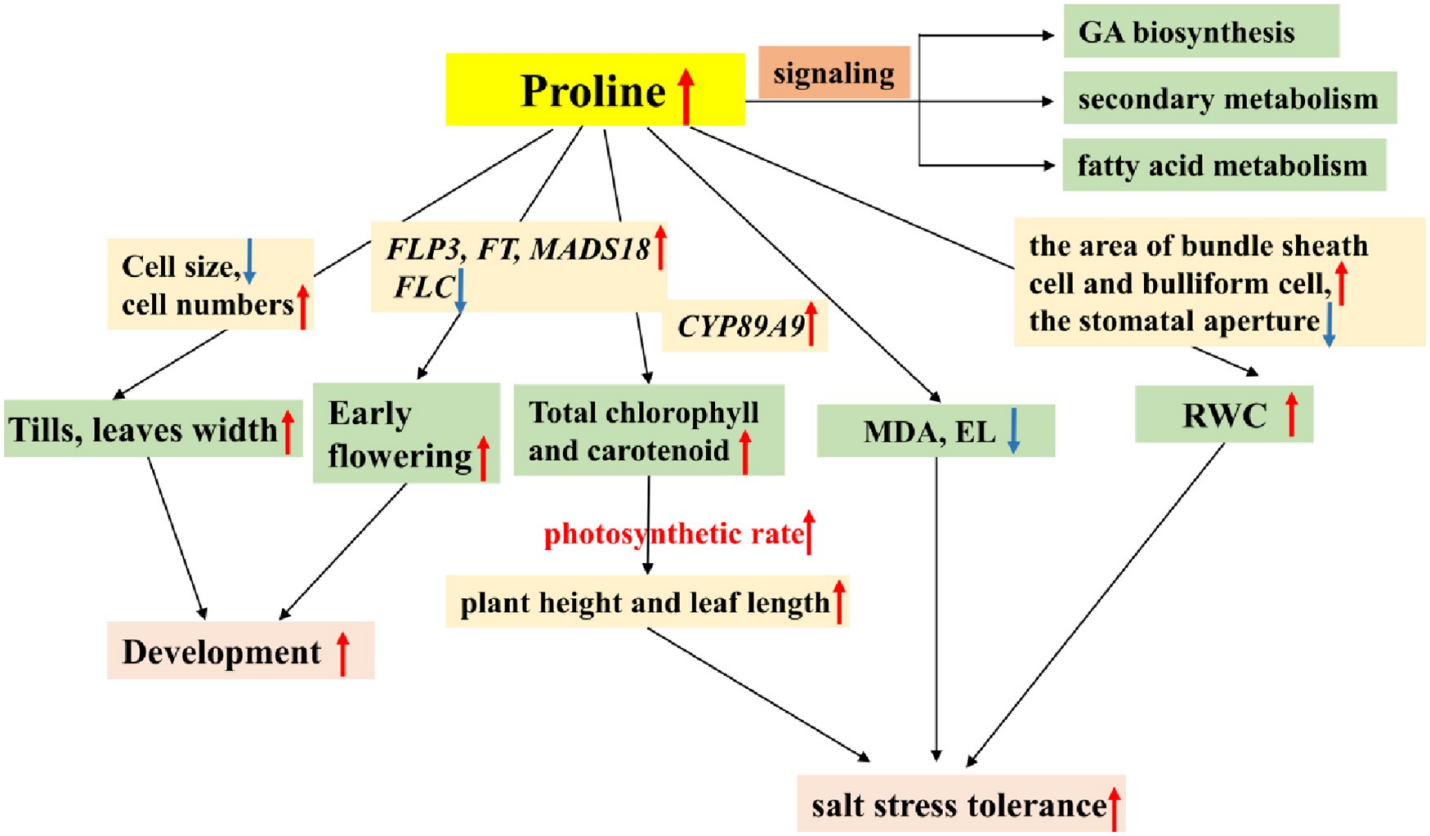
Proline coordinated events and pathways to regulate plant growth and development and salt stress tolerance.

Proline can protect and stabilize ROS scavenging enzymes and activate alternative detoxification pathways, which led to improved salt tolerance (Fig 9). Recent paper reports that proline metabolism is regulated by fatty acid synthesis in Arabidopsis [10]. Our data of RNA-seq also showed that deficient expression of *P5CS* affected the secondary metabolism and fatty acid metabolism pathways. In conclusion, biomass and stress tolerance are the two important traits required for switchgrass as an energy crop. Obviously, group I transgenic lines showed a significant potential for genetic improvement of biomass production and salt tolerance in switchgrass, and field evaluation experiments are required to confirm the greenhouse data.

## Supporting information

S1 Fig Homogeneous analysis. (TIF)

S2 Fig Molecular identification. (A) PCR and (B) Southern blot identification in transgenic plants.
(TIF)

S3 Fig Correlations of expression levels analyzed by log_2_RNA-Seq platform with data obtained using log_2_qPCR. X-axis: log_2_RNA-Seq; Y-axis: log_2_qPCR.
(TIF)

S4 Fig Relative expression levels of *PvCYP89A9*, *PvCYP714B3*, *PvCYP78A6*, *PvCYP97B2*, *PvCYP86A2*, *PvCYP72A14*, *PvCYP94A1* and *PvCYP88A1* in transgenic and WT plants. Switchgrass *Ubq1* was used as the reference for normalization. Value are mean ± SE (*n* = 6). The significance of treatments was tested at the *P* < 0.05 level (one way ANOVA, Dunnett’s test).
(TIF)

S5 Fig Distribution of transcription-factor of differentially expressed transcripts in transgenic and WT switchgrass. (TIF)

S6 Fig Preliminary evaluation of salt tolerance in transgenic and WT plants. (A) The result of the preliminary experiment of *in vitro* leaves from transgenic and WT plants under salt stress on the 30^th^ day, (B) transgenic lines were treated with 350 mM NaCl solution for a week, (C) RWC and (D) EL in transgenic and WT plants under 350 mM NaCl solution for a week. Value are mean ± SE (*n* = 3). The significance of treatments was tested at the *P* < 0.05 level (one way ANOVA, Dunnett’s test).
(TIF)

S7 Fig Biomass of transgenic and WT plants under salt stress. (A) The fresh and (B) dry weight of transgenic and WT plants under 0, 200 and 400 mM NaCl concentration. The significance of treatment (0, 200 and 400 mM NaCl concentration) an sample type (WT and transgenic plants) was tested at the *P* < 0.05 level (two way ANOVA), capital letter represents the difference between WT and transgenic plants under the same salt concentration, lowercase represents the difference of WT or transgenic plants under 0, 200 and 400 mM NaCl concentration.
(TIF)

S1 Table Primers used in this study. (DOCX)

S2 Table Summary statistics of Illumina transcriptome sequencing. (DOCX)

S3 Table excel The most significantly up-regulated differentially expressed genes (log2Fold Change *>* 2). (XLSX)

S4 Table excel The most significantly down-regulated differentially expressed genes (log2Fold Change *>* 2). (XLSX)

S5 Table excel GO enrichment. (XLSX)

S6 Table excel KEGG enrichment. (XLSX)

S7 Table excel The differentially expressed transcription factors (TFs). (XLSX)
