# Supplementary figures and images for: Overexpression of the Lolium perenne L. delta1-pyrroline 5-carboxylate synthase (LpP5CS) gene results in morphological alterations and salinity tolerance in switchgrass (Panicum virgatum L.)
Source: PLoS One. 2019 Jul 16;14(7):e0219669. doi: 10.1371/journal.pone.0219669 (PMC6634860; doi:10.1371/journal.pone.0219669)

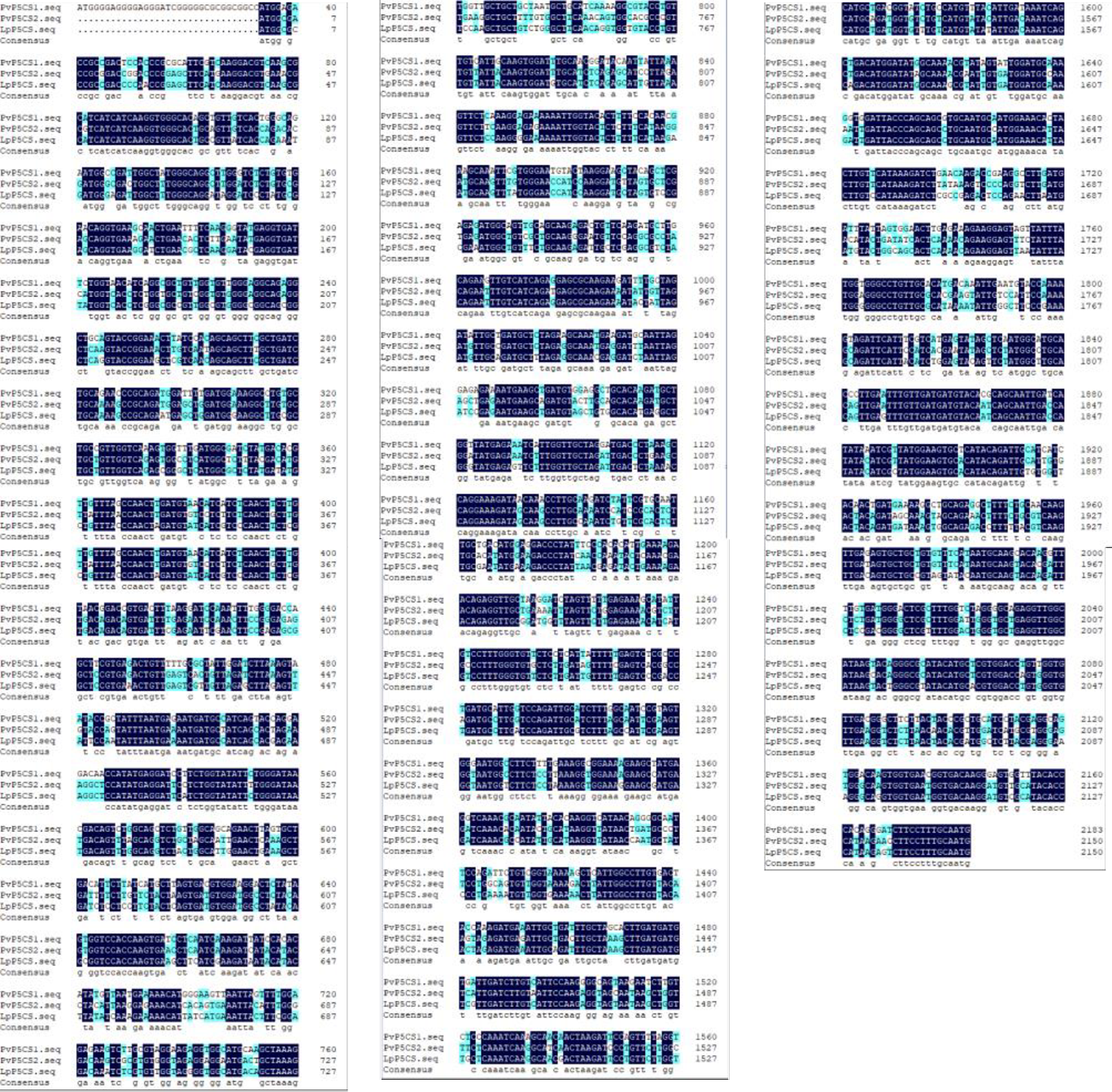

Supplement: S1 Fig — (TIF) [file pone.0219669.s001.tif]

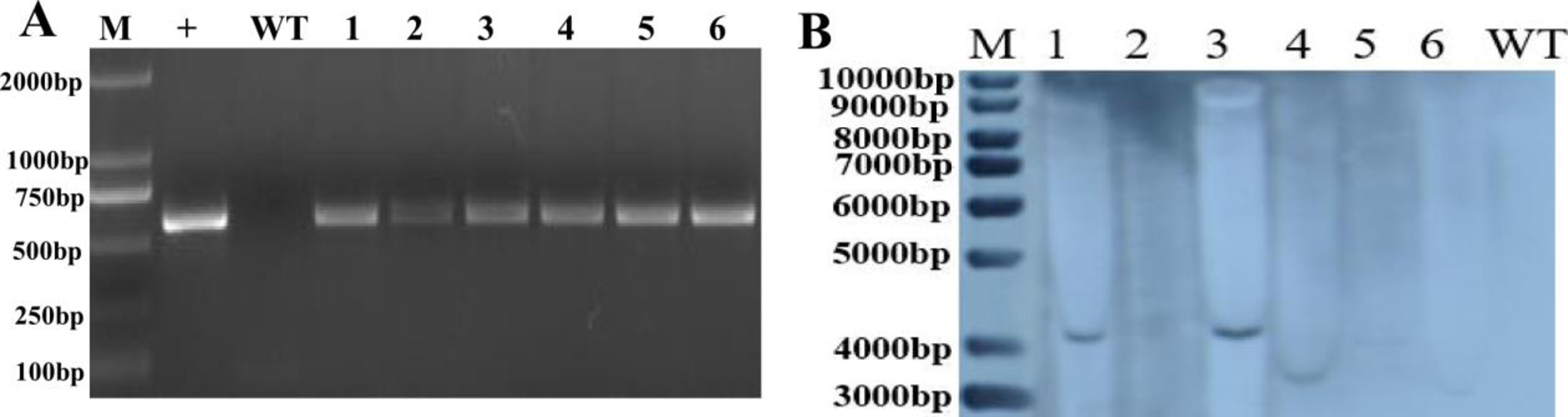

Supplement: S2 Fig — (A) PCR and (B) Southern blot identification in transgenic plants. (TIF) [file pone.0219669.s002.tif]

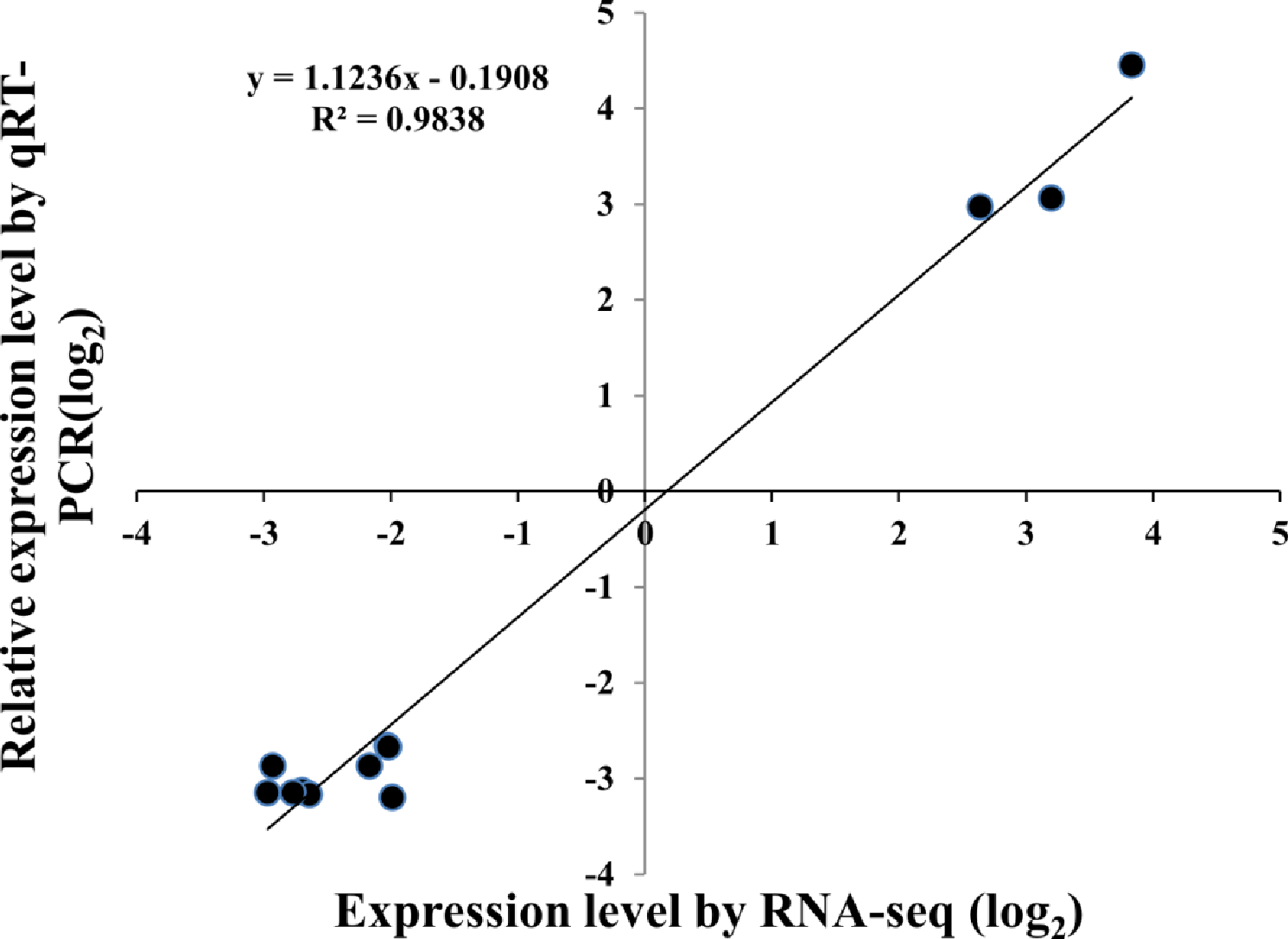

Supplement: S3 Fig — X-axis: log2RNA-Seq; Y-axis: log2qPCR. (TIF) [file pone.0219669.s003.tif]

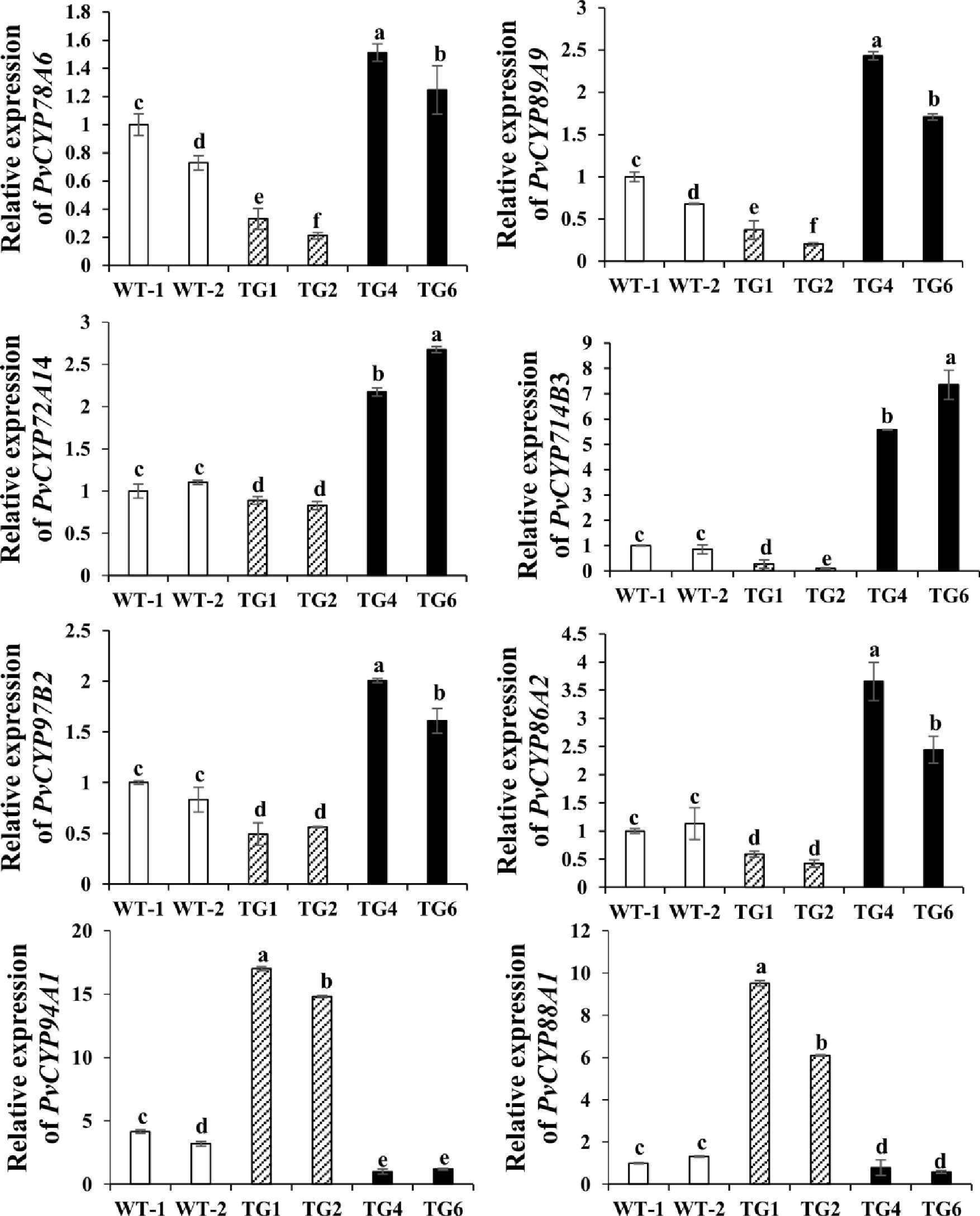

Supplement: S4 Fig — Switchgrass Ubq1 was used as the reference for normalization. Value are mean ± SE (n = 6). The significance of treatments was tested at the P < 0.05 level (one way ANOVA, Dunnett’s test). (TIF) [file pone.0219669.s004.tif]

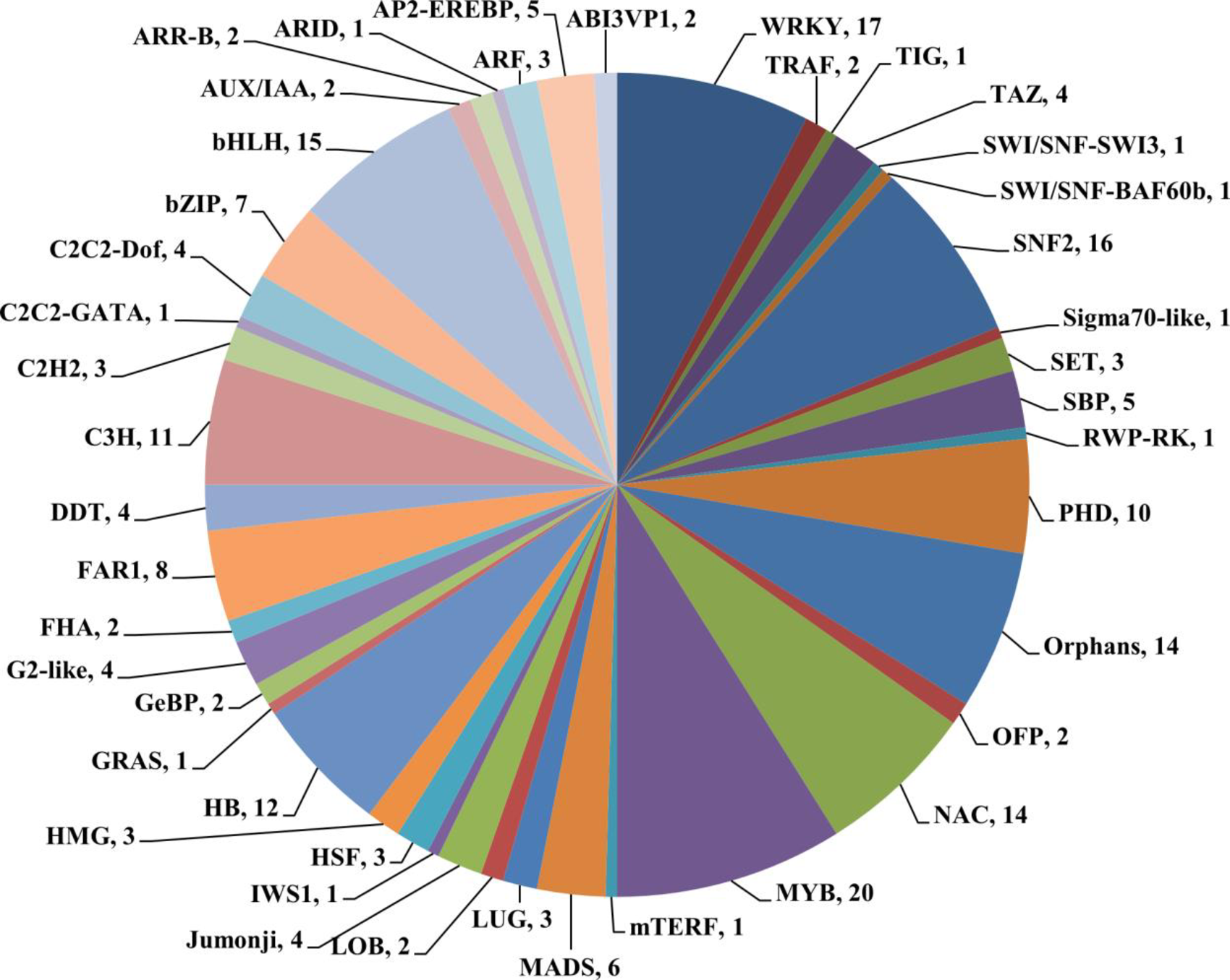

Supplement: S5 Fig — (TIF) [file pone.0219669.s005.tif]

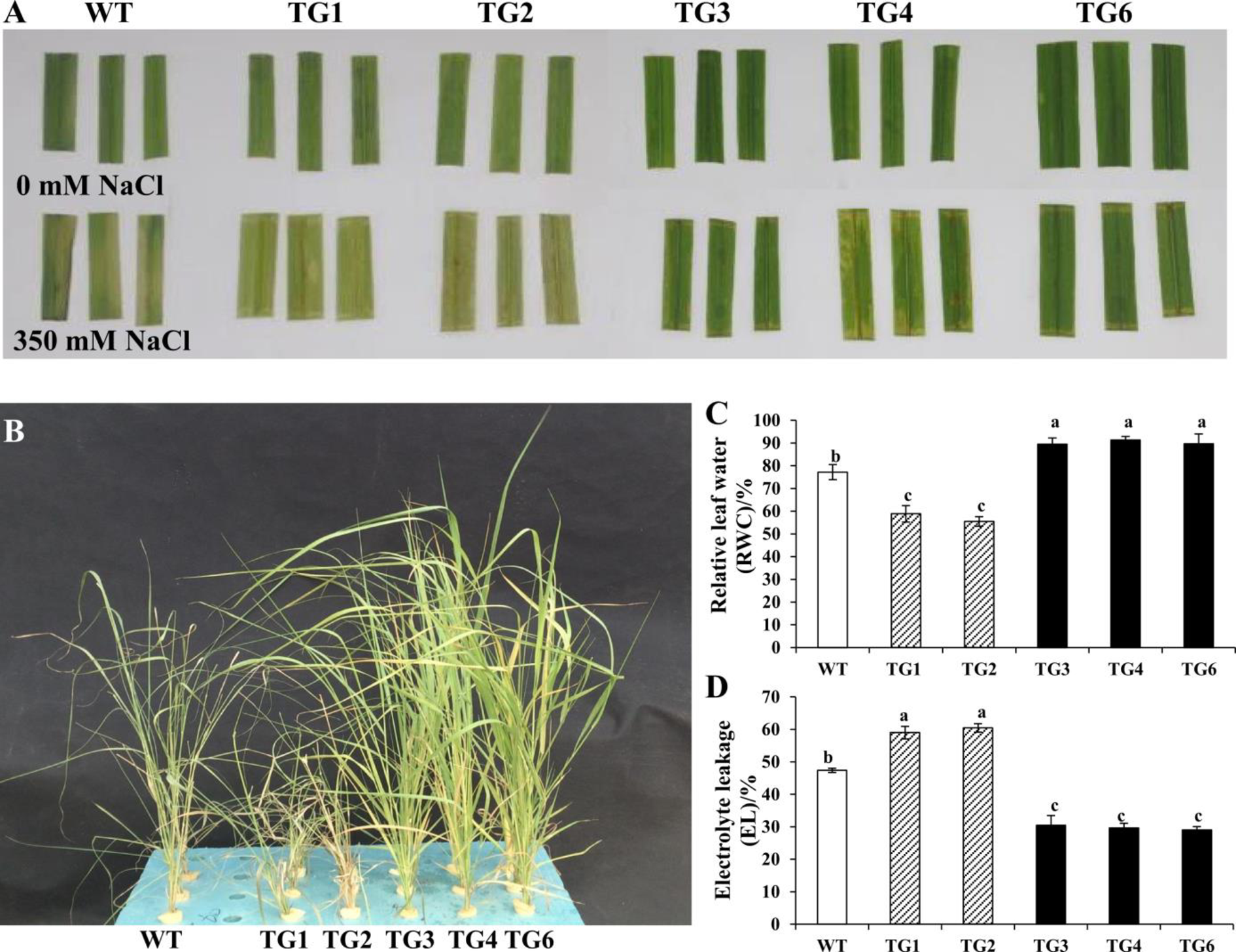

Supplement: S6 Fig — (A) The result of the preliminary experiment of in vitro leaves from transgenic and WT plants under salt stress on the 30th day, (B) transgenic lines were treated with 350 mM NaCl solution for a week, (C) RWC and (D) EL in transgenic and WT plants under 350 mM NaCl solution for a week. Value are mean ± SE (n = 3). The significance of treatments was tested at the P < 0.05 level (one way ANOVA, Dunnett’s test). (TIF) [file pone.0219669.s006.tif]

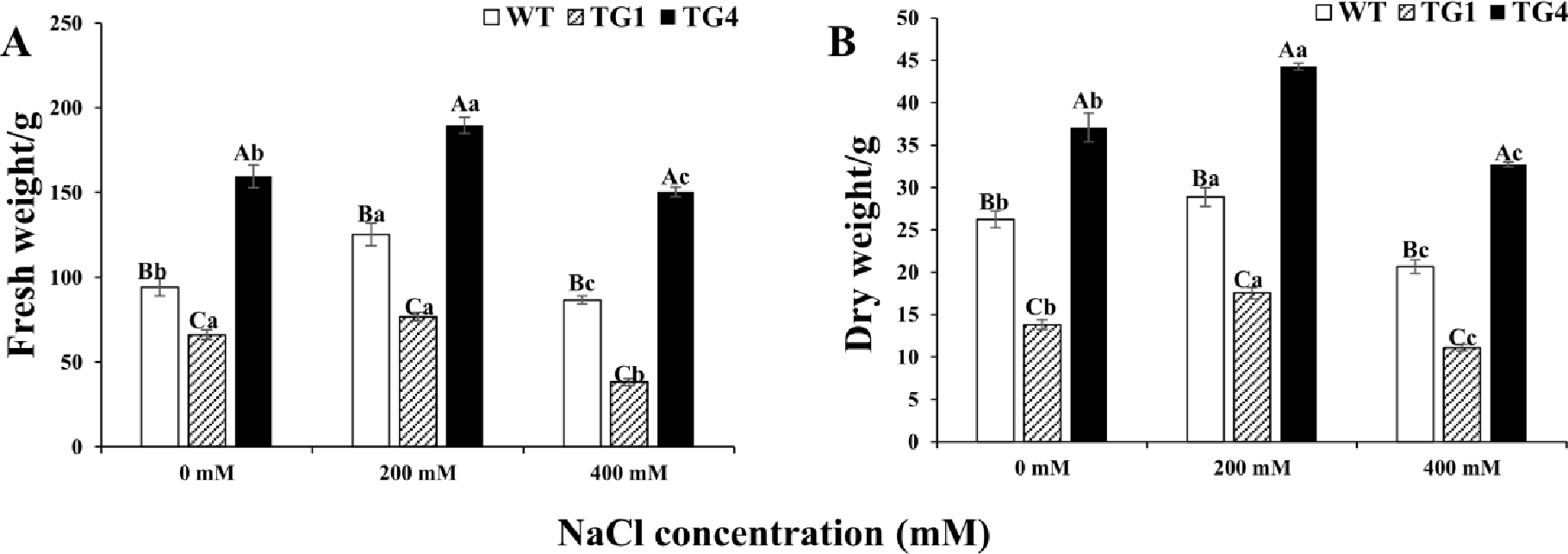

Supplement: S7 Fig — (A) The fresh and (B) dry weight of transgenic and WT plants under 0, 200 and 400 mM NaCl concentration. The significance of treatment (0, 200 and 400 mM NaCl concentration) an sample type (WT and transgenic plants) was tested at the P < 0.05 level (two way ANOVA), capital letter represents the difference between WT and transgenic plants under the same salt concentration, lowercase represents the difference of WT or transgenic plants under 0, 200 and 400 mM NaCl concentration. (TIF) [file pone.0219669.s007.tif]
