# Supplementary material for: Overexpression of the Lolium perenne L. delta1-pyrroline 5-carboxylate synthase (LpP5CS) gene results in morphological alterations and salinity tolerance in switchgrass (Panicum virgatum L.)
Source: PLoS One. 2019 Jul 16;14(7):e0219669. doi: 10.1371/journal.pone.0219669 (PMC6634860; doi:10.1371/journal.pone.0219669)
Supplement: S1 Table — (DOCX) [file pone.0219669.s008.docx]

**S1 Table Primers used in this study**

| **Name/Accession no.** | **Sequence (5’-3’)** | **Purpose** |
| --- | --- | --- |
| ***LpP5CS* (KC896627)**  ***LpP5CS* (KC896627)**  ***PvUBIQUITIN1* (****FL955474.1)**  ***PvP5CS1* (Pavir.J02344.1)**  ***PvP5CS2***  **(Pavir.J06546)**  ***PvProDH***  **( Pavir.Ia02415.1)**  ***PvCYP89A9***  **(RNA seq_23805544)**  ***PvCYP714B3***  **(RNA seq_23776646)**  ***PvCYP78A6***  **(RNA seq_23809399)**  ***PvCYP97B2***  **(RNA seq_23760156)**  ***PvCYP86A2***  **(RNA seq_23765424)**  ***PvCYP88A1***  **(RNA seq_23790946)**  ***PvCYP724B1***  **(RNA seq_23802413)**  ***PvCYP98A1***  **(RNA seq_23767096)**  ***PvFLP5***  **(RNA seq_23807914)**  ***PvMADS18***  **(RNA seq_23802753)**  ***PvSPL9***  **(RNA seq_23816442)**  ***PvFT***  **(RNA seq_23783191)**  ***PvMADS14***  **(RNA seq_23757063)**  ***PvFLP3***  **(RNA seq_23758014)**  ***PvCYP72A14***  **(RNA seq_23776238)**  ***PvCYP94A1***  **(RNA seq_23808148)**  ***PvCAT***  **(Pavir.J03636.1)**  ***PvSOD***  **(Pavir.Ib01670.1)**  ***PvGST***  **(Pavir.Ib02422.1)**  ***PvPOD***  **(Pavir.Eb02543.1)**  ***PvCYCD***  **(Pavir.J14518.1)**  ***PvCYCB***  **(Pavir.J38704.1)** | F1: TCTGAACGCTCAAGGATACG  R1: ATGCCACCCCTACCAACACG  F2: AACCGGAGCTTCATCAAGGA  R2: ATCCTTCACCTGCTCGCATA  F3: CAGCGAGGGCTCAATAATTCCA  R3: TCTGGCGGACTACAATATCCA  F4: GACCCTAAAGCCAGGAAAGATAA  R4: CTAGATCCTTAGCAACCTCTGTTC  F5: TAGGTCATGCTGACGGTATCT  R5: TGCTGGGTAATCCACCTTTG  F6: GCGTACCGCCACTTCTG  R6: CGTCCTCGATCCCGTAGT  F7: CACCAGATTCTCCACGAATGAC  R7: CGGCAGAACGTTGAAGATGA  F8: GCCGAGGCAGAGAACTTTAT  R8: TGCAGTCCAAGGAGCATTAG  F9: GCGTGTGTGGGAGAAGATAG  R9: ACAGGAGTATACACAGGACAGTA  F10: AGCAAAGGGACTACACATCTC  R10: CCAGCAATAAGCATCGTCATAAG  F11: CTTCGAGAAGCACGACCTC  R11: CAGTAGGAAGCTGATCACGAC  F12: GCTGAGAGACTTCAGGAAGATG  R12: CAGGTACCCGTTCACAAAGA  F13: GGATCATCTCCCTCCCTATCA  R13: CGATGAACTTCTCCATGGTCTC  F14: GGACGTCACCTACAAAGGATAC  R14: CATCTGCGAGGCTGAAACT  F15: GTGTACGTGCCGACGAA  R15: GATGCTTCGGTTCTCGTAGTAG  F16: GCTCCTCTGCACCATCATATT  R16: TACAGCATACATCTCCAACCAC  F17: CACTCAAACAAGACCGGAGAT  R17: CAGCGGTAAGTGACCATAACA  F18: CCTGTGTTTGATGGGATGGA  R18: CTGCGAAGTGATGGCTTACTA  F19:GAACGCTACTCCTATGCAGAAA  R19:TGTCTCAACCTTCGCCTTTAG  F20:CGCTACCTCTACGATCCCT  R20:GTACATGTGGACGAGCTTGA  F21: GTGAGAAATGGGCAAAGCATAG  R21: CGATGCAACAGGTAGCAAATAC  F22:GGCAGAGGCAGGGTAATTAAA  R22:GAAAGACTGGGCTCGAGATG  F23:CATGCTGAGAAGGTTCCTATCC  R23:ACCAGCCTGCTTGAAGTTATT  F24:TGTCAACTGGACCGCATTAC  R24:AGTTAGCAACACCATCATCTCC  F25:ATCCGCGTCGACTCCTT  R25:TTGTACTTGTCCGCGATGTG  F26:GAGGTCATCGACGAGATCAAG  R26:CAGTCATGACGGTGGAGTC  F27:CAAGACGACAAGGAGGAAGATAC  R27:CTCGGAAGCAGCCATTAATCT  F28:GCTAGTGGGAGTAACAGCTATG  R28:AATCTGCCCTTTCGTGTAGG | DNA confirmation of *LpP5CS* for transgenic identification  Quantitative expression of *LpP5CS*  Quantitative expression  of *PvUBIQUITIN1*  Quantitative expression of *PvP5CS1*  Quantitative expression of *PvP5CS2*  Quantitative expression of  *PvProDH*  Quantitative expression of *PvCYP89A9*  Quantitative expression of  *PvCYP714B3*  Quantitative expression of  *PvCYP78A6*  Quantitative expression of *PvCYP97B2*  Quantitative expression of *PvCYP86A2*  Quantitative expression of *PvCYP88A1*  Quantitative expression of *PvCYP724B1*  Quantitative expression of *PvCYP98A1*  Quantitative expression of *PvFLP5*  Quantitative expression of *PvMADS18*  Quantitative expression of *PvSPL9*  Quantitative expression of  *PvFT*  Quantitative expression of  *PvMADS14*  Quantitative expression of  *PvFLP3*  Quantitative expression of  *PvCYP72A14*  Quantitative expression of  *PvCYP94A1*  Quantitative expression of  *PvCAT*  Quantitative expression of *PvSOD*  Quantitative expression of *PvGST*  Quantitative expression of *PvPOD*  Quantitative expression of *PvCYCD*  Quantitative expression of *PvCYCB* |
