# Supplementary material for: Overexpression of the Lolium perenne L. delta1-pyrroline 5-carboxylate synthase (LpP5CS) gene results in morphological alterations and salinity tolerance in switchgrass (Panicum virgatum L.)
Source: PLoS One. 2019 Jul 16;14(7):e0219669. doi: 10.1371/journal.pone.0219669 (PMC6634860; doi:10.1371/journal.pone.0219669)
Supplement: S2 Table — (DOCX) [file pone.0219669.s009.docx]

**S2 Table Summary statistics of Illumina transcriptome sequencing**

|  | **Wild-type (WT)** | |  | **Group II transgenic line (TG)** | |  | **Group I transgenic line (TG)** | |
| --- | --- | --- | --- | --- | --- | --- | --- | --- |
|  | WT1 | WT2 |  | TG1 | TG2 |  | TG4 | TG6 |
| **Raw reads** | 71599346 | 70459624 |  | 71689978 | 70962912 |  | 76439496 | 64305834 |
| **Clean reads** | 70282168 | 69123202 |  | 70322428 | 69567180 |  | 75201562 | 62832784 |
| **clean bases** | 10.54G | 10.37G |  | 10.55G | 10.44G |  | 11.28G | 9.42G |
| **Error rate (%)** | 0.01 | 0.01 |  | 0.01 | 0.01 |  | 0.01 | 0.01 |
| **Q20 (%)** | 98.32 | 97.75 |  | 98.07 | 98.1 |  | 98.36 | 97.81 |
| **Q30 (%)** | 95.85 | 94.67 |  | 95.3 | 95.38 |  | 95.9 | 94.63 |
| **GC content (%)** | 53.09 | 54.15 |  | 55.29 | 53.58 |  | 54.56 | 54.16 |
